# EGFR and HER2 expression in primary cervical cancers and corresponding lymph node metastases: Implications for targeted radiotherapy

**DOI:** 10.1186/1471-2407-8-232

**Published:** 2008-08-12

**Authors:** Li Shen, Yongjie Shui, Xiaojia Wang, Liming Sheng, Zhengyan Yang, Danfeng Xue, Qichun Wei

**Affiliations:** 1 Department of Radiation Oncology, the Second Affiliated Hospital, Zhejiang University School of Medicine, Hangzhou 310009, PR China; 2 Ministry of Education Key Laboratory of Cancer Prevention and Intervention, Zhejiang University School of Medicine, Hangzhou 310009, PR China; 3 Zhejiang Cancer Hospital, Banshan, Hangzhou, PR China

## Abstract

**Background:**

Proteins overexpressed on the surface of tumor cells can be selectively targeted. Epidermal growth factor receptor (EGFR) and human epidermal growth factor receptor 2 (HER2) are among the most often targeted proteins. The level and stability of expression in both primary tumors and corresponding metastases is crucial in the assessment of a receptor as target for imaging in nuclear medicine and for various forms of therapy. So far, the expression of EGFR and HER2 has only been determined in primary cervical cancers, and we have not found published data regarding the receptor status in corresponding metastatic lesions. The goal of this study was to evaluate whether any of these receptors are suitable as target for clinical diagnosis and therapy.

**Methods:**

Expression of EGFR and HER2 was investigated immunohistochemically in both lymph node metastases and corresponding primary cervical cancers (n = 53). HER2 and EGFR expression was scored using HercepTest criteria (0, 1+, 2+ or 3+).

**Results:**

EGFR overexpression (2+ or 3+) was found in 64% (35/53) of the primary cervical tumors and 60% (32/53) of the corresponding lymph node metastases. There was a good concordance between the primary tumors and the paired metastases regarding EGFR expression. Only four patients who had 2+ or 3+ in the primary tumors changed to 0 or 1+ in lymph node metastases, and another two cases changed the other way around. None of the primary tumors or the lymph node metastases expressed HER2 protein.

**Conclusion:**

The EGFR expression seems to be common and stable during cervical cancer metastasis, which is encouraging for testing of EGFR targeted radiotherapy. HER2 appears to be of poor interest as a potential target in the treatment of cervical cancer.

## Background

Cervical cancer represents the second most frequent malignancy in women worldwide, particularly in developing countries [1]. While curable in early stages, the prognosis for advanced stage disease is poor [2]. Radiation has been the gold standard of therapy for many decades. Nowadays, concurrent cisplatin-based chemoradiotherapy has been considered as the standard therapeutic modality for locally advanced cervical cancer [3-6]. However, such treatment remains suboptimal with histopathological residual tumor observed in 40–50% of patients [7,8]. Those presenting with recurrent or metastatic disease have limited treatment options [2,9], and the 5-year survival is less than 5% [10]. There is a clear need for novel, more effective therapeutical strategies to improve overall survival and the quality of life for advanced, recurrent and disseminated cervical cancer. Thus, the testing of molecular targeted therapies against cervical cancer is a logical step to follow [11]. Another strategy is receptor-mediated tumor targeted radiotherapy [12], which is based on the delivery of therapeutically relevant radionuclides directly to disseminated tumor cells, with hopefully minimal damage to normal tissues.

Proteins overexpressed on the surface of tumor cells can be selectively targeted. Epidermal growth factor receptor (EGFR) and human epidermal growth factor receptor 2 (HER2) are among the most often targeted proteins. The presence of EGFR and HER2 receptors have been associated with accelerated tumor progression and therapeutic resistance for several types of malignancies, including cervical cancer. The causal relationship of EGFR and HER2 receptor network to disease progression and resistance to therapy provides a rationale for therapeutic intervention.

Nowadays, EGFR targeted drugs, both chimeric monoclonal antibody Cetuximab (Erbitux) and the small-molecule tyrosine kinase inhibitors (e.g. Iressa and Tarceva) [13-15], have been approved by FDA. Clinical trials of Cetuximab, either alone or in combination with radiotherapy/chemotherapy, have recently demonstrated efficacy in patients with head and neck cancer, colorectal cancer and lung cancer [16-18]. The humanized monoclonal antibody trastuzumab (Herceptin), which is the first clinically available oncogene-targeted therapeutic agent for treatment of solid tumors, has boosted the interest of physicians in targeting therapy, as therapeutic benefit was proved in patients with HER2-positive metastatic breast cancer [19]. On the basis of the preliminary success, clinical trials are currently investigating the therapeutic potential of molecular targeted drugs in other human malignancies including cervical cancer [20].

In a recent phase II trial reported by Goncalves *et al*, patients with recurring locoregionally advanced or metastatic cervical cancer were treated with gefitinib 500 mg/day, EGFR expression levels by means of immunohistochemistry did not correlate with tumor response and disease control [20]. This is not unexpected, since EGFR expression does not necessarily correlate with EGFR receptor activation. Other molecular alterations, such as EGFR gene amplification, mutations of the tyrosine kinase domain, and EGFR phosphorylation status, might be useful indicators for the response to EGFR signaling inhibition. However, in the case of targeted radionuclide therapy, tumor cells are mainly killed with delivered radiation and therapeutic efficiency is only dependent on the receptor expression and not whether the receptor function can be blocked or not. Thus, receptor overexpression is considered necessary for the success of targeted radiotherapy.

Previous studies have shown EGFR to be frequently expressed in primary cervical cancer [21-25], and that EGFR expression is correlated with poor prognosis [25-27]. Positive staining of HER2 in cervical cancer has been reported to vary wildly in the earlier studies [28-30]. However, according to the recent reports using HercepTest grading system, HER2 overexpression was considered to be a rare event in primary cervical cancer [31,32].

The literature was reviewed for similar investigations, but no other studies comparing primary cervical cancers and their corresponding metastases regarding EGFR and HER2 were found by the authors. It is still unclear whether the metastases lose, gain, or retain the receptor status relative to the primary tumors. For a receptor to be of interest for targeting, similar expression in both the primary tumors and the disseminated lesions are required. Investigation into the receptor status between metastases and the primary tumors will provide valuable information on whether these receptors are suitable as target for diagnostic and/or therapeutic procedures. In the present study, the expression of EGFR and HER2 was investigated immunohistochemically in a series of 53 primary cervical cancers and corresponding lymph node metastases.

## Methods

### Patients and Samples

Patients with cervical cancer who were treated with radical hysterectomy and systematic pelvic lymphadenectomy between 2002 and 2003, were enrolled in the present study. Tumor samples from all patients were obtained at the time of operation through the Gynecologic Oncology Department and the Pathology Department, Zhejiang Cancer Hospital, under ethical approval of the Institutional Review Board of Zhejiang Cancer Hospital. Informed consent for scientific evaluation had been obtained from patients. Paraffin sections from both the primary tumors and the corresponding lymph node metastases were required for inclusion. Tissue samples were not taken from distant metastases so these were not available for analysis. Totally, 53 patients with high quality material were finally included in the study. Clinical information was obtained from the hospital records and included patient age, disease stage, histological pattern, differentiation, tumor size, nodal involvement, lymphatic/vascular invasion, vaginal invasion, parametrial invasion, ovarian metastasis. All patients had a pathologic diagnosis of squamous cell carcinoma. Ovarian metastasis was not found in this set of cases. The patient and tumor characteristics of the analyzed cases are shown in Table 1.

**Table 1:** Tumor and patient characteristics (n = 53)

Characteristics	Patients, n (%)
Differentiation	
High	5 (9.4)
Moderate	42 (79.3)
Low	6 (11.3)
	
FIGO Stage	
IB	16 (30.2)
IIA	35 (66.0)
IIB	1 (1.9)
IIIB	1 (1.9)
	
Tumor size	
> 5 cm	9 (17.0)
3–5 cm	26 (49.0)
< 3 cm	18 (34.0)
	
Lymphatic or vascular space invasion	
Positive	26 (49.1)
Negative	27 (50.9)
	
Vaginal invasion	
Positive	37 (69.8)
Negative	16 (30.2)
	
Parametrial invasion	
Positive	6 (11.3)
Negative	47 (88.7)
	
Age (years)	
Medium	48
Range	29–72

Briefly, the tissues were fixed in 4% buffered formalin, processed and embedded in paraffin. Sections, 4-μm thick, were then cut and deparaffinized in xylene and hydrated through graded concentrations of ethanol to distilled water.

### EGFR-staining

EGFR was assessed by immunohistochemistry using a streptavidin-biotin complex technique as previously described [33]. After deparaffinization of the sections, endogenous peroxidase was blocked in 0.3% H_2_O_2 _in PBS for 20 min. For antigen retrieval, the sections were submitted to high temperature and pressure with Tris-EDTA buffer (pH 9) for 5 min. The slides were preincubated in PBS for 10 min. The primary mouse monoclonal antibody directed against EGF receptor (clone 31G7, Zymed labs, South San Francisco, CA, USA) receptor were diluted 1:100, and incubated overnight at 4°C. The secondary biotinylated antibodies (goat anti-mouse from Dako, Glostrup, Denmark) and the peroxidase-labelled streptavidin-biotin complex (Dako) were diluted 1:200 and incubated for 30 min at room temperature. All slides were developed in 0.05% diamino benzidine (Sigma, St Louis, MO, USA) for 5 min and counterstained in Harris haematoxylin (Sigma). Finally, the slides were dehydrated through graded alcohol to xylene and mounted in organic mounting medium.

### HER2-staining

The HER2 immunohistochemical staining was made as previously described [34]. After deparaffinization, the sections were incubated in methanol and hydrogen peroxide for 30 min quenching endogenous peroxidase. Antigen retrieval was done in waterbath at 98°C, pH 6 for 40 minutes. Thereafter the slides were cooled at room temperature and then washed in distilled water. Immunohistochemical staining was performed using the Elite ABC Kit (Vectastain, Vector Laboratories, Burlingame, CA). Blocking serum was applied for 15 min and followed by incubation with rabbit anti-human c-erbB-2 oncoprotein (code No. A 0485, Dako) diluted 1:350. Sections were then incubated with the biotinylated secondary antibody and were visualised by using the peroxidase substrate 3-amino-9-ethyl-carbazole (AEC) (Sigma A-5754) as chromogen. Finally, the sections were counterstained with Mayer's haematoxylin and mounted.

### EGFR and HER2-scores

The HER2 expression was scored using the HercepTest scoring criterion. The HER2-score was based on a scale where 0 corresponded to tumor cells that were completely negative, 1+ corresponded to faint perceptible staining of the tumor cell membranes, 2+ corresponded to moderate staining of the entire tumor cell membranes and 3+ was strong circumferential staining of the entire tumor cell membranes creating a fishnet pattern. The Canadian and the DAKO HercepTest guidelines were applied, which require more than 10% of the tumor cells to be stained [35]. Cytoplasmic staining was considered non-specific and was not included in the scoring. As positive controls we used in house positive control tissue sections as well as positive control sections supplied by DAKO. As negative controls we used normal tissues, which are expected not to express HER2 such as connective tissue seen in the same sections as the tumor cells. In the metastases sections we used lymphocytes and the surrounding capsule of the lymph nodes as negative internal controls. The expression pattern of EGFR is quite similar to that of HER2, and EGFR expression was therefore evaluated using the same scoring criterion as for HER2. As EGFR positive controls we used in house positive control skin tissue sections. As negative controls we used connective tissue seen in the same sections as the tumor cells. In the metastases sections we used lymphocytes and the surrounding capsule of the lymph nodes as negative internal controls.

## Results

### Expression of EGFR

**Figure 1:**
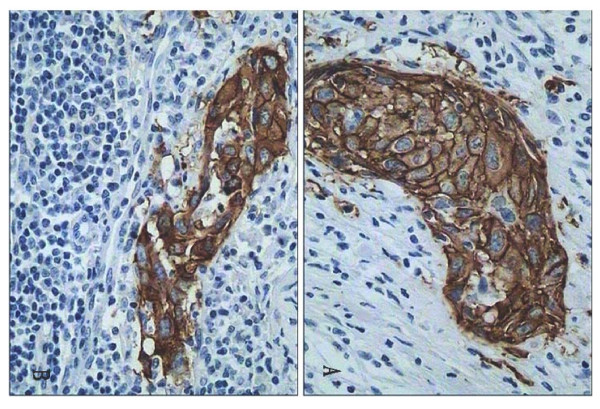
**Comparisons of immunohistochemical EGFR staining of primary cervical carcinoma (A) and corresponding metastases (B).** Both A and B (from the same patient) were scored 3+. The micrographs were taken with objective × 40.

Table 2 shows the EGFR-scores for the analyzed 53 primary cervical squamous cell carcinoma and the corresponding 53 lymph node metastases. EGFR overexpression (2+ or 3+) was found in 64% (34/53) of the primary cervical tumors and 60% (32/53) of the corresponding lymph node metastases. There was a good agreement between the primary tumors and the corresponding lymph node metastases in the majority of cases. Fifteen changes were observed. However, there were only four patients who had 2+ or 3+ in the primary tumors and changed to 0 or 1+ in lymph node metastases, and another two patients who had 0 or 1+ in the primary tumors and changed to 2+ or 3+ in lymph node metastases. Examples of staining patterns for a primary tumor and the corresponding metastasis (which both were scored as 3+) are shown in Figure 1A and 1B.

**Table 2:** EGFR-scores for the analyzed primary cervical squamous cell carcinoma and the corresponding lymph node metastases (n = 53)

Primary tumor EGFR-scores	Lymph node metastases EGFR-scores			
	0	1+	2+	3+
0	5	1	0	0
1+	1	10	2	0
2+	0	3	13	3
3+	1	0	4	10

The scoring was based on a scale where 0 corresponded to completely negative staining, 1+ corresponded to faint perceptible staining of the tumor cell membranes, 2+ corresponded to moderate staining of the entire tumor cell membranes and 3+ was strong circumferential staining of the entire tumor cell membranes creating a fishnet pattern

### Expression of HER2

**Figure 2:**
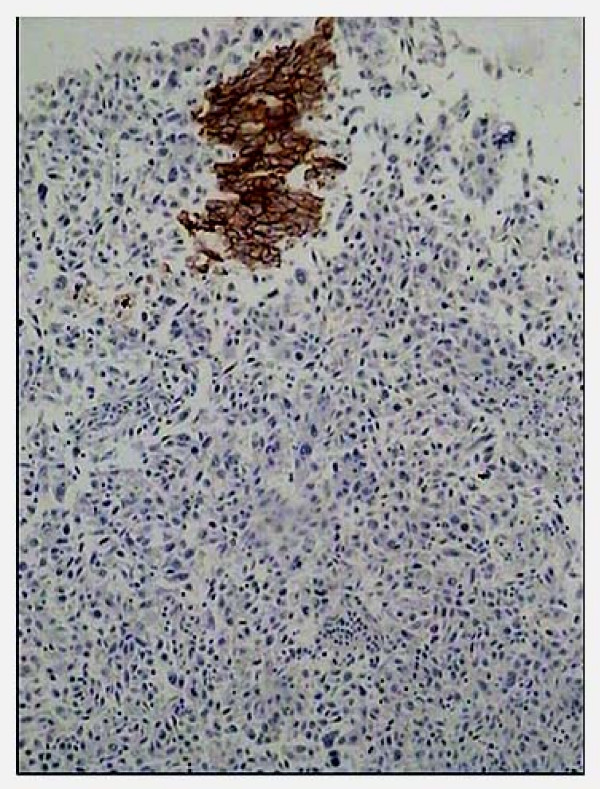
**Example of immunohistochemical HER2 staining of primary cervical carcinoma, scored 0 (stained cells were less than 10% of total tumor cells).** This case also had negative HER2 staining in lymph node metastasis. The micrograph was taken with objective × 10.

Among the 53 paired samples, none of the primary tumors or the lymph node metastases expressed HER2 protein. In fact, a few strong membrane stained tumor cells were seen in 2 cases, but the stained cells were less than 10% of total tumor cells (Figure 2). So, these two cases were also interpreted as negative.

## Discussion

The aim of this study was to evaluate the EGFR and HER2 receptor expression, using immunohistochemical analyses, in primary cervical cancers and determine if the expression is retained in lymph node metastases. The goal was to evaluate whether any of these receptors are suitable as target for radionuclide based imaging and radiation therapy.

Overexpression of EGFR in cervical cancer has been reported to be common (ranges from 26–72%) [21-25]. However, it is unclear whether metastases lose, gain, or retain EGFR status relative to the primary cervical tumors. Studies on the EGFR status of metastatic lymph node of cervical cancer will provide precious knowledge to evaluate whether the receptor is of interest for diagnostic and/or therapeutic procedures or not.

EGFR overexpression (2+/3+) was found in 64% of the primary lesions. Our result is consistent with the former findings of high EGFR overexpression in cervical cancer [23-25]. Furthermore, we found that the frequency of EGFR overexpression in lymph node metastases was approximately as high as in the primary lesions of cervical cancer. Although 15 changes were observed, only 4 patients with EGFR overexpression in the primary tumor had lower EGFR scores in the corresponding lymph node metastases. Moreover, in another two patients, EGFR overexpression was gained in lymph node metastases while the primary tumors had low scores. Actually, immunohistochemistry is not a strickly quantitative method. Methodological differences, e.g. in fixation procedures, retrieval methods, and antibodies, are likely to affect the sensitivity of EGFR staining. In addition, the criteria for defining positive EGFR expression could also account for part of the discrepancies between the primaries and metastases. For example, if membrane staining in more than 10% of the tumor cells was defined as positive EGFR expression, regardless of pattern of cellular membrane staining (complete or incomplete), only 3 changes could be observed: two cases from positive to negative when the primary lesions were compared to the corresponding lymph node metastases, and one case changed the other way around. Nevertheless, it seems that, in a majority of the studied cases, EGFR expression retained in the metastases.

To our knowledge, the question of EGFR status in lymph node metastases versus primary cervical cancer has not been addressed. From our results, it seems that EGFR expression is stable when comparing the lymph node metastases with the primary cervical cancers, which is surprising in the light of the genomic instability that characterizes most malignant tumors. Although the current report is limited by the small sample size, our observations suggest that EGFR expression in the primary tumors, which can readily be determined after surgery or biopsy, might predict EGFR-positive metastases with a reasonably high probability.

In EGFR targeted radionuclide therapy, possible side effects to normal tissues should be taken into consideration, as EGFR is commonly expressed in normal cells. It might be possible to minimize the toxicity and improve therapeutic efficiency by using suitable targeting agents with low uptake in critical normal tissues, and suitable biodistribution. EGFR targeted radiotherapy might also be possible if a tumor and its metastases have a strong EGFR expression to ensure higher tumor uptake than in most normal tissues or local delivery of the targeting agent can be made.

There are conflicting results regarding the frequency of HER2 expression in cervical cancer. High positive rate of HER2 expression in cervical cancer has been reported in the earlier studies [28-30]. For example, Lee *et al *[28] found HER2 expression in 29.7% of the analyzed cases. Nevin *et al *[29] and Niibe *et al *[30] reported HER2 expression in about 40% of the studied cases. However, according to the recent reports by Chavez-Blanco *et al *[31] and Kim *et al *[32], HER2 expression was observed in only 3.2% (1/31) and 0% (0/227), respectively, of the studied squamous cell cervical cancer cases. The original aim of the present study was to compare HER2 expression between the primary uterine cervical tumors and the corresponding lymph node metastases. Surprisingly, none of the cases expressed HER2 protein, neither in the primary lesions nor in the metastases. The use of positive controls during immunohistochemical procedures rules out the hypothesis of false-negative reactions. The application of HercepTest scoring criterion, the official FDA scoring guidelines for predictive assessment in breast cancer, might be an important reason for the low positive rate of HER2 expression. Using the standardized HercepTest scoring criterion, HER2 expression is considered a rare event in cervical cancer [31,32]. Our data are consistent with these findings. Therefore, HER2 appears to be of poor interest as a potential target in the treatment of cervical cancer.

## Conclusion

The present study is the first to compare the EGFR and HER2 receptor status in primary cervical cancers with their lymph node metastases. For a receptor to be of interest for targeting nuclides therapy, similar expression in both the primary tumors and the disseminated lesions are required. The EGFR expression seems to be common and stable during cervical cancer metastasis, which is encouraging for testing of EGFR targeted radiotherapy. HER2 appears to be of poor interest as a potential target in the treatment of cervical cancer.

## Competing interests

The authors declare that they have no competing interests.

## Authors' contributions

LS participated in the design of the study, carried out the clinical and immunohistochemical data analysis; YS and LS interpreted the histological and immunohistochemical data; XW, ZY and DX contribute with the clinical data; and QW conceived the study, interpreted the immunohistochemical data and wrote the manuscript. All authors read and approved the final manuscript.

## Pre-publication history

The pre-publication history for this paper can be accessed here:


